# Potential Locations for Noninvasive Brain Stimulation in Treating Poststroke Deficits: Exploration From Multimodal Neuroimaging

**DOI:** 10.1002/cns.71229

**Published:** 2026-10-29

**Authors:** Sitong Yuan, Yue Yang, Yi Han, Ying Gao, Jingling Chang, Huize Lin, Jinna Yu, Binlong Zhang

**Affiliations:** ^1^ Department of Acupuncture and Neurology, Guang'anmen Hospital China Academy of Chinese Medical Sciences Beijing China; ^2^ Shandong First Medical University & Shandong Academy of Medical Sciences Jinan China; ^3^ Department of Neurology, Dongzhimen Hospital Beijing University of Chinese Medicine Beijing China

**Keywords:** multimodal neuroimaging, noninvasive brain stimulation, stimulation target, stroke

## Abstract

**Aims:**

Noninvasive brain stimulation (NIBS) shows promise for poststroke deficits (PSDs), but efficacy may be limited by inconsistent stimulation sites.

**Methods:**

By integrating neuroimaging meta‐analysis, lesion‐symptom mapping (LSM), and resting‐state functional connectivity (FC) analysis, we employed three analytical pipelines to explore potential NIBS targets for alexia, aphasia, apraxia, ataxia, dysarthria, hemiplegia, paresthesia, and dysphagia. Pipeline 1 identified cortical regions directly associated with each PSD via coordinate‐based meta‐analysis using Neurosynth Compose. Pipeline 2 identified cortical regions exhibiting maximal functional connectivity with neurosynth‐derived regions of interest (ROI). Pipeline 3 identified cortical regions functionally connected to ROIs from a prior large‐scale LSM study. During Pipeline 2 and 3, ROIs were used for seed‐to‐voxel FC analysis using resting‐state fMRI data from 100 healthy subjects. Identified cortical targets were mapped onto the scalp and referenced by the 10–20 system and continuous proportional coordinates.

**Results:**

Potential cortical targets and scalp locations were identified for all eight PSDs. For instance, for hemiplegia, validated targets included primary motor and somatosensory cortices and supplementary motor area; novel targets included angular gyrus, superior frontal gyrus, inferior parietal lobule, medial prefrontal cortex, and precuneus.

**Conclusions:**

This multimodal approach systematically identified precise NIBS targets, confirming established sites and revealing novel regions to broaden neuromodulation strategies for poststroke recovery.

## Graphical abstract

We developed a three‐pipeline framework combining meta‐analysis, lesion‐symptom mapping, and functional connectivity to identify cortical targets for noninvasive brain stimulation (NIBS) across eight poststroke deficits. This approach systematically identifies precise NIBS targets, confirms established sites, and reveals novel regions to broaden neuromodulation strategies for poststroke recovery.

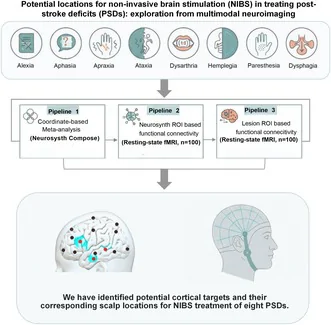

## 1 Introduction

Stroke is characterized by an acute focal injury to the central nervous system due to vascular causes, including cerebral infarction, intracerebral hemorrhage, and subarachnoid hemorrhage [1]. It has high rates of incidence, recurrence, disability, and mortality, leading to generally poor prognoses [2]. Following neurological injury, 50%–70% of stroke survivors experience poststroke deficits (PSD) such as hemiplegia, dysphagia, sensory disorders, cognitive impairment, hemianopia, aphasia, alexia, and apraxia, which significantly reduce patients' quality of life and place a heavy burden on their families and society [3]. Therefore, as current options remain limited, there is growing interest in effective therapeutic methods that can improve neurological dysfunction.

Recently, noninvasive brain stimulation (NIBS) has been applied in the treatment of PSDs. This technology primarily includes repetitive transcranial magnetic stimulation (rTMS), transcranial direct current stimulation (tDCS), and transcranial alternating current stimulation (tACS). Studies have confirmed the potential of NIBS in the treatment of PSDs [4]. However, recent meta‐analyses on the efficacy of these NIBS therapies remain controversial. Inconsistencies in stimulation sites may lead to variations in efficacy, requiring further research and refinement of NIBS sites.

In recent years, multimodal neuroimaging techniques, especially magnetic resonance imaging (MRI), have undergone rapid development. These imaging techniques can simultaneously acquire information on the precise structure of brain regions, the trajectory and integrity of white matter fiber tracts, and the functional state of cerebral gray matter. This has broadened the understanding of brain regions and brain networks related to various poststroke dysfunctions, deeply revealing the evolution of connection and cooperation patterns between brain regions after stroke, and leading to a more profound understanding of the pathological and recovery mechanisms of stroke [5, 6]. Within neuroimaging research, stroke is distinguished from other disorders by its characteristic focal brain lesions. This neuropathological signature positions stroke as an ideal model for investigating region‐specific brain functions. By implementing structural MRI‐based lesion‐symptom mapping (LSM), researchers can directly elucidate causal relationships between stroke lesions and PSDs [7, 8].

This study therefore aimed to explore potential NIBS targets for different PSDs. Building upon our team's established methodology of multimodal neuroimaging meta‐analysis and functional connectivity for identifying neuromodulation targets in neuropsychiatric disorders [9, 10, 11], we now integrate LSM to incorporate stroke‐specific neuropathology, enabling precision targeting of PSDs.

## 2 Methods

Three pipelines combining neuroimaging meta‐analysis, lesion‐symptom mapping, functional connectivity, and cortex‐scalp mapping were applied to explore potential NIBS targets for different PSDs. We first obtained PSD‐related ROIs using results from meta‐analysis and LSM. Subsequently, based on resting‐state fMRI data from 100 healthy subjects, these ROIs were utilized for seed‐to‐voxel functional connectivity analysis to identify cortical regions most strongly associated with each PSD. Finally, these cortical regions were mapped onto the scalp surface to identify potential NIBS targets (Figure 1).

**FIGURE 1:**
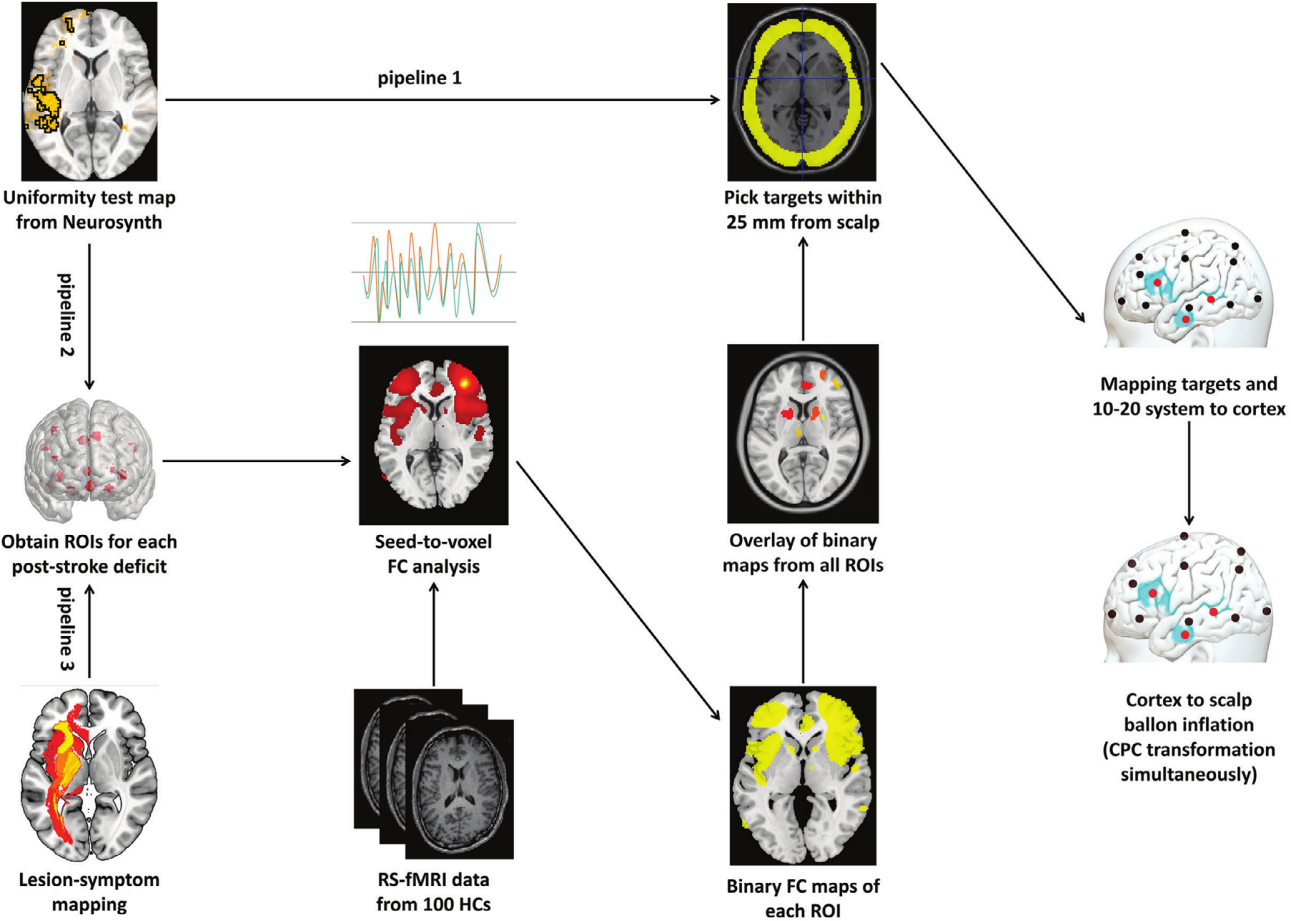
Flowchart of the three data processing pipelines for identifying potential NIBS targets in PSDs. In Pipeline 1, potential cortical clusters were selected directly from the neurosynth‐derived uniformity test map. In Pipeline 2, clusters were explored from FC results based on the ROIs obtained from Neurosynth‐based meta‐analysis. In Pipeline 3, clusters were explored from FC results based on the ROIs obtained from LSM analysis. Finally, cortical clusters and coordinates were mapped onto scalp and referenced by 10–20 system and CPC coordinates. CPC, continuous proportional coordinates; FC, functional connectivity; HC, healthy controls; LSM, lesion‐symptom mapping; PSD, poststroke deficits; ROI, region of interest; RS‐fMRI, resting‐state functional MRI. The background anatomical image used in this figure is the ch2bet.nii standard brain template provided by the DPABI software.

### 2.1 Identify PSD‐Associated Brain Regions From Meta‐Analysis

Neurosynth Compose (https://compose.neurosynth.org/), a web‐based meta‐analysis platform, was used to identify brain regions associated with different PSDs [12]. For eight PSDs (hemiplegia, paresthesia, aphasia, alexia, apraxia, dysphagia, dysarthria, and ataxia), we formulated tailored search terms and performed neuroimaging literature searches on July 26, 2024. Adhering to PRISMA guidelines, two independent researchers implemented a four‐phase process: identification, screening, eligibility assessment, and inclusion, culminating in finalized study selection. Studies were excluded based on the following criteria: non‐stroke investigations, studies not focusing on the relevant PSDs, animal research, pediatric studies, review articles, and literature lacking standard MNI space coordinates. Subsequent procedures included coordinates extraction and coordinate‐based meta‐analysis (CBMA). The meta‐analysis algorithm employed multilevel kernel density analysis (MKDA) with chi‐square analysis under FDR correction (alpha of 0.05). Similar to our previous study [10], the uniformity test map was applied to identify brain regions consistently activated across studies for each PSD.

### 2.2 Identify PSD‐Associated Brain Regions From Lesion‐Symptom Mapping

We utilized LSM results from a recent large‐scale multicenter study involving 7807 stroke patients [7]. This study correlated subitem scores of the National Institutes of Health Stroke Scale (NIHSS) with lesion locations through LSM analysis, identifying peak‐associated lesion areas for each PSD. From the lesion maps of voxel‐based LSM, we extracted the most significantly associated lesion coordinates for: hemiplegia (NIHSS items 5a, 5b, 6a, 6c), ataxia (NIHSS item 7), paresthesia (NIHSS item 8), aphasia (NIHSS item 9), and dysarthria (NIHSS item 10). The coordinates (Table S1) were then used to construct ROIs for functional connectivity analysis.

### 2.3 Building ROIs for Functional Connectivity Analysis

Two distinct ROI sets were constructed for functional connectivity analysis: (1) Meta‐analytic ROIs: A predetermined cutoff of the top ten highest *z*‐score coordinates was extracted from the Neurosynth uniformity test maps for each PSD. This threshold was selected to balance analytic granularity with interpretability, avoiding spatial redundancy from numerous local maxima within large clusters while aligning with common resting‐state network practices (typically five to fifteen nodes per network); (2) LSM‐derived ROIs: coordinates from prior LSM results. To ensure anatomical and physiological validity during the resting‐state functional connectivity analysis, any peak coordinates localized within white matter tracts were systematically excluded. Only coordinates localized to cortical and subcortical gray matter were retained. Spherical masks (6‐mm radius) centered on these coordinates were generated using MarsBaR v0.45 (https://marsbar‐toolbox.github.io/). Meta‐analytic ROIs underwent refinement via overlap between spherical masks and original uniformity test maps to ensure only voxels in the uniformity test maps were included.

### 2.4 Participants and MRI Acquisition

Resting‐state fMRI (RS‐fMRI) and three‐dimensional structural T1‐weighted MRI (3D‐T1) data of 100 healthy subjects were obtained from the Southwest University Adult Lifespan Dataset (SALD) (https://fcon_1000.projects.nitrc.org/indi/retro/sald.html) [13]. The mean age was 60.3 years (standard deviation [SD] 3.8 years), and 30 patients (30.0%) were women, who were age‐ and gender‐matched with the participants used in the LSM study [7]. SALD provides anonymized and publicly accessible MRI data with basic phenotypic information requiring no additional ethical approval. All original studies associated with the database had obtained necessary ethics committee approvals and documented informed consent prior to data collection. Specific acquisition parameters are available in Appendix S1.

### 2.5 Image Preprocessing

Similar to previous studies [9, 10], after removing the first 10 volumes, the RS‐fMRI data of the 100 subjects were processed in SPM 12 (https://www.fil.ion.ucl.ac.uk/spm/software/spm12/) and Conn version 22a using Conn's default preprocessing pipeline (https://web.conn‐toolbox.org/) [14]. During preprocessing, functional images underwent slice‐timing correction, realignment, normalization (3 × 3 × 3 mm^3^), smoothing (6 × 6 × 6 mm^3^), outliers detection (> 3 SD and > 0.5 mm), linear regression (white matter signal, cerebrospinal fluid signal, linear trend, subject motion, and outliers scrubbing), and band‐pass filtering (0.01–0.1 Hz).

### 2.6 Functional Connectivity Analysis

Using the ROIs identified from the meta‐analysis, seed‐to‐voxel FC analyses were performed. Residual BOLD time courses were extracted from each subject's ROIs to compute subject‐level seed‐to‐voxel connectivity maps. Voxel‐wise Pearson correlation coefficients between seed regions and all brain voxels were calculated, followed by Fisher's *z*‐transformation to satisfy normality assumptions for subsequent group‐level analyses. Group connectivity patterns were identified through one‐sample *t*‐tests on all individual *z*‐transformed connectivity maps. Group‐level correlation maps were then generated using a voxel‐wise level threshold of *p* < 0.001 and a cluster level family‐wise error (FWE) of *p* < 0.05.

### 2.7 Exploring Brain Surface Targets for PSDs

Three different pipelines were conducted to explore potential brain surface targets for PSDs. Given that the electrical field generated by NIBS reduces as the cortex‐scalp distance increases, we employed a 25‐mm depth mask from the scalp surface for target selection to ensure NIBS accessibility to brain surface targets [9, 15].

#### 2.7.1 Pipeline 1

Using the 25 mm brain surface mask, potential cortical clusters were selected directly from the Neurosynth‐derived uniformity test map. During cluster selection, the *T*‐value threshold on the map was progressively raised in 0.5 increments until 4–6 cortical clusters with cluster sizes of 30–800 voxels were identified. When no qualifying clusters were found that strictly met both criteria simultaneously (e.g., yielding fewer than 4 clusters or exceeding the 800‐voxel limit), an appropriate statistical threshold was collectively determined through author consensus. For instance, in Pipeline 1 for apraxia (*T* = 4.5), only 2 valid clusters remained; in Pipeline 2 for dysarthria (*T* = 0, positive correlation), the maximum cluster size reached 1163 voxels. Manual threshold adjustments through author consensus were specifically required for aphasia, apraxia, paresthesia, and dysphagia in Pipeline 1; alexia, dysarthria, paresthesia, and dysphagia in Pipeline 2; and aphasia, ataxia, dysarthria, hemiplegia, and paresthesia in Pipeline 3. These clusters may represent cortical regions directly involved in the pathophysiology of each PSD.

#### 2.7.2 Pipeline 2

Thresholded group‐level correlation maps for each Neurosynth‐derived ROI were converted to binary masks. A third‐level correlation map was generated by overlaying these binary masks (separately for positive/negative correlations), where voxel intensity in the map represents the count of ROIs functionally connected to each voxel. Cortical clusters were then identified from the third‐level map using the same protocol as Pipeline 1. These clusters likely represent cortical regions exhibiting maximal functional connectivity with PSD‐associated networks.

#### 2.7.3 Pipeline 3

Pipeline 3 followed analogous procedures to Pipeline 2, with the exception that the refined, gray‐matter‐exclusive LSM‐derived ROIs were utilized to generate binary masks and subsequent third‐level correlation maps. The cortical clusters identified through this pipeline represent both: (1) the neuroanatomically relevant surface regions physiologically linked to PSD lesions and (2) the brain areas most susceptible to functional reorganization post‐stroke.

Following cortical cluster identification, target coordinates were selected within each cluster. For Pipeline 1, peak *T*‐value MNI coordinates in the clusters were identified. For Pipelines 2 and 3, cluster centroid coordinates were computed as the mean MNI coordinate of all voxels within a cluster using xjView (www.alivelearn.net/xjview).

### 2.8 Validation Using Poststroke Patient Data

To address the potential limitation that functional connectivity patterns in healthy brains may not fully reflect the reorganized networks of a poststroke brain, a supplementary validation analysis was conducted. We utilized resting‐state fMRI data from an independent cohort of 23 poststroke patients with hemiplegia (mean age: 67.3 years). The data acquisition parameters, preprocessing steps, and seed‐to‐voxel functional connectivity analyses were performed identically to the primary pipelines described above. Specifically, both Meta‐derived ROIs (Pipeline 2) and LSM‐derived ROIs (Pipeline 3) for hemiplegia were used as seeds to generate patient‐specific group‐level correlation maps. This allowed us to verify whether the core spatial topology of the targeted networks was conserved in the pathological state.

### 2.9 Mapping Clusters and Coordinates From Cortex to Scalp

Cortical clusters and coordinates were mapped on a standard brain using Surf Ice (www.nitrc.org/projects/surfice/), referenced by the 10–20 system in MNI space [16]. Then, the clusters and coordinates were mapped onto the scalp through a balloon inflation. In addition, the coordinates were transformed to continuous proportional coordinates (CPC) as Pnz‐Pal parameters using MNI2CPC (https://transcranial‐brain‐atlas.org/mni2cpc/). This neuro‐navigation‐free approach facilitates precise scalp targeting of cortical regions, with validated efficacy across diverse demographic and pathological populations [15, 17].

## 3 Result

After identifying ROIs from LSM (Table S1) and meta‐analysis (Tables S2 and S3), we explored potential targets for alexia, aphasia, apraxia, ataxia, dysarthria, hemiplegia, paresthesia, and dysphagia from three pipelines. Detailed 10–20 system locations and CPC coordinates for all identified regions are presented in Tables 1, 2, 3 and visualized in Figures 2, 3, 4.

**TABLE 1:** Results of Pipeline 1.

Pipeline 1		Cluster size	MNI coordinates			Brain regions	10–20 system locations	CPC coordinates	Depth
			*x*	*y*	*z*			(Pnz, Pal)	
Aph	aph1	337	−46	22	20	BA.L	Inferior and posterior to F3	0.32, 0.28	22.82
	aph2	43	−58	−12	−24	MTG.L	Inferior to T3	0.44, 0.09	23.54
	aph3	63	−56	−42	0	WA.L	Anterior to T5	0.73, 0.17	23.28
Ale	ale1	49	−54	−6	18	M1/S1.L	Inferior to C3	0.44, 0.21	24.97
	ale2	305	62	−34	4	pSTG.R	Posterior and superior to T4	0.64, 0.84	19.57
	ale3	165	64	−6	−6	mSTG.R	Anterior to T4	0.40, 0.86	17.57
	ale4	64	−22	−94	−6	PVC.L	Anterior and inferior to O1	0.94, 0.40	18.61
	ale5	175	−42	−78	−18	VAC.L	Inferior to O1	0.95, 0.29	19.02
Apr	apr1	143	−56	−36	28	SMG.L	Anterior and inferior to P3	0.63, 0.22	22.84
	apr2	47	−54	−44	−8	pMTG.L	Anterior to T5	0.77, 0.16	24.59
Hem	hem1	162	−48	0	36	M1.L	Anterior and inferior to C3	0.43, 0.29	22.56
	hem2	637	−38	−22	54	M1.L	Posterior and superior to C3	0.52, 0.35	24.71
	hem3	564	0	−4	68	SMA	Proximal to Cz	0.47, 0.50	24.75
	hem4	747	38	−18	54	M1.R	Posterior and superior to C4	0.50, 0.65	24.72
Par	par1	63	−58	−22	40	S1/SMG.L	Posterior and inferior to C3	0.54, 0.26	18.08
	par2	31	42	−46	52	SPL.R	Anterior to P4	0.64, 0.68	23.75
	par3	202	54	−26	38	S1/SMG.R	Posterior and inferior to C4	0.55, 0.74	23.19
Dysp	dysp1	87	−58	−12	12	S1/M1.L	Superior to T3	0.48, 0.18	22.62
	dysp2	68	58	−6	8	S1/M1.R	Anterior and superior to T4	0.43, 0.82	22.98

Abbreviations: Ale, alexia; Aph, aphasia; Apr, apraxia; BA, Broca's area; CPC, continuous proportional coordinate; Dysp, dysphagia; Hem, hemiplegia; L, left; M1, primary motor cortex; MNI, Montreal Neurological Institute; MTG, middle temporal gyrus; Par, paresthesia; PVC, primary visual cortex; R, right; S1, primary somatosensory cortex; SMA, supplementary motor area; SMG, supramarginal gyrus; SPL, superior parietal lobule; STG, superior temporal gyrus; VAC, visual association cortex; WA, Wernicke's area.

**TABLE 2:** Results of Pipeline 2.

Pipeline 2		Cluster size	MNI coordinates			Brain regions	10–20 system locations	CPC coordinates	Depth
			*x*	*y*	*z*			(Pnz, Pal)	
Aph‐neg	aphm4	85	−60	−3	−13	MTG.L	Anterior to T3	0.37, 0.13	20.61
	aphm6	41	−41	−78	9	TOJ.L	Superior and anterior to O1	0.86, 0.32	19.93
	aphm8	87	52	−59	23	ANG.R	Superior to T6	0.75, 0.74	20.57
	aphm9	208	60	−3	−17	MTG.R	Inferior and anterior to T4	0.34, 0.88	21.16
	aphm10	137	4	65	−14	mPFC	Inferior and anterior to Fp2	0.09, 0.51	18.81
Aph‐pos	aphm1	103	−39	38	20	MFG.L	Inferior and posterior to F3	0.26, 0.32	20.01
	aphm2	65	−39	45	−5	MFG.L	Anterior to F7	0.16, 0.30	21.04
	aphm3	209	−53	15	7	BA.L	Superior and posterior to F7	0.31, 0.21	22.46
	aphm5	35	−52	−45	42	IPL.L	Anterior to P3	0.65, 0.28	21.25
	aphm7	34	−1	32	50	dmPFC	Posterior to Fz	0.34, 0.49	24.62
Ale‐neg	alem4	82	20	54	25	MFG.R	Inferior and anterior to F4	0.22, 0.58	19.67
	alem5	64	51	−41	53	IPL.R	Superior and anterior to P4	0.61, 0.69	17.88
Ale‐pos	alem1	41	−48	−63	−15	IOG.L	Inferior to T5	0.90, 0.22	23.77
	alem2	80	−34	−80	22	ANG/MOG.L	Inferior and posterior to P3	0.83, 0.36	20.53
	alem3	106	38	−77	25	MOG.R	Inferior and posterior to P4	0.81, 0.66	19.4
Apr‐neg	aprm4	48	−36	−60	53	IPL.L	Superior and anterior to P3	0.70, 0.36	21.27
	aprm5	255	−34	−75	37	IPL.L	Inferior and posterior to P3	0.79, 0.37	19.83
	aprm7	187	7	−63	63	PCUN.R	Anterior to Pz	0.69, 0.52	21.79
	aprm8	548	40	−68	41	ANG.R	Proximal to P4	0.75, 0.66	19.52
	aprm11	144	29	26	56	SFG.R	Superior and posterior to F4	0.37, 0.61	15.65
	aprm12	71	43	21	46	MFG.R	Superior and posterior to F4	0.37, 0.66	15.46
Apr‐pos	aprm1	300	−53	6	15	BA/M1.L	Inferior and anterior to C3	0.38, 0.22	23.78
	aprm2	149	−60	−23	28	SMG/S1.L	Inferior and posterior to C3	0.55, 0.22	19.58
	aprm3	51	−60	−41	23	SMG.L	Superior and anterior to T5	0.66, 0.21	18.9
	aprm6	233	2	2	68	SMA	Inferior and anterior to Cz	0.45, 0.50	23.03
	aprm9	50	59	−19	32	SMG/S1.R	Inferior and posterior to C4	0.52, 0.76	20
	aprm10	33	58	5	16	M1.R	Superior and anterior to T4	0.38, 0.79	20.38
Dysa‐neg	dysam4	84	−35	−70	44	ANG.L	Proximal to P3	0.76, 0.37	20.21
Dysa‐pos	dysam1	356	−30	45	24	MFG.L	Proximal to F3	0.25, 0.36	20.31
	dysam2	1076	−52	2	25	M1.L	Inferior and anterior to C3	0.41, 0.25	23.2
	dysam3	215	−57	−32	23	SMG.L	Superior and anterior to T5	0.61, 0.21	22.92
	dysam5	1163	−3	−4	70	SMA	Proximal to Cz	0.47, 0.49	22.98
	dysam6	838	54	7	20	M1.R	Inferior and anterior to C4	0.37, 0.76	22.8
	dysam7	137	32	43	26	MFG.R	Inferior and anterior to F4	0.26, 0.64	20.6
Hem‐neg	hemm4	79	0	−68	57	PCUN.R	Proximal to Pz	0.72, 0.50	22.86
	hemm6	397	42	−62	47	ANG.R	Superior and anterior to P4	0.72, 0.66	19.53
	hemm8	46	28	20	62	SFG.R	Superior and posterior to F4	0.39, 0.60	14.59
	hemm9	35	55	−58	37	ANG.R	Inferior and anterior to P4	0.71, 0.72	15.82
Hem‐pos	hemm1	90	−34	−10	63	M1.L	Superior to C3	0.49, 0.37	18.54
	hemm2	60	−59	−11	11	M1/S1.L	Superior to T3	0.47, 0.18	21.63
	hemm3	32	−3	−1	68	SMA	Anterior to Cz	0.46, 0.48	23.79
	hemm5	64	27	−46	66	S1.R	Posterior and inferior to Cz	0.62, 0.60	22.25
	hemm7	651	41	−6	55	M1.R	Superior to C4	0.47, 0.66	19.61
	hemm10	149	60	−8	12	M1/S1.R	Superior to T4	0.45, 0.82	20.99
Par‐neg	parm4	220	−43	−66	41	ANG.L	Proximal to P3	0.75, 0.33	19.08
Par‐pos	parm1	164	−55	5	23	M1.L	Inferior and anterior to C3	0.40, 0.24	20.46
	parm2	33	−21	−8	66	PMA.L	Superior to C3	0.48, 0.41	22.17
	parm3	1187	−53	−31	43	S1.L	Posterior to C3	0.57, 0.28	21.8
	parm5	50	−53	−65	−6	TOJ.L	Proximal to T5	0.86, 0.23	19.09
	parm6	65	54	−56	−4	TOJ.R	Proximal to T6	0.81, 0.80	21.78
	parm7	511	54	9	24	M1.R	Posterior to C4	0.37, 0.75	21.07
	parm8	385	25	−4	65	PMA.R	Superior and anterior to C4	0.47, 0.59	20.86
	parm9	1886	48	−32	49	S1.R	Inferior and anterior to C4	0.57, 0.69	23.07
Dysp‐neg	dyspm1	497	−35	17	55	PMA.L	Superior and anterior to C3	0.40, 0.37	15.49
	dyspm2	1083	−36	−67	43	IPL.L	Proximal to P3	0.75, 0.36	22.02
	dyspm3	35	15	41	51	PMA.R	Inferior and anterior to Fz	0.31, 0.56	16.84
	dyspm4	192	−2	−70	57	PCUN.L	Proximal to Pz	0.73, 0.49	21.49
	dyspm5	1038	42	−64	43	IPL.R	Proximal to P4	0.73, 0.67	19.95
	dyspm6	120	63	−34	−11	MTG.R	Posterior to T4	0.68, 0.87	18.97
	dyspm9	904	34	22	52	PMA.R	Superior and posterior to F4	0.37, 0.63	17.07
Dysp‐pos	dyspm7	269	49	−5	49	M1.R	Proximal to C4	0.46, 0.69	17.7
	dyspm8	81	60	3	12	M1.R	Superior and anterior to T4	0.38, 0.80	19.29

*Note:* pos, positive: brain surface regions positively correlated with the PSDs ROIs; neg, negative: brain surface regions negatively correlated with the PSD ROIs, CPC continuous proportional coordinate, L left, R right.

Abbreviations: Ale, alexia; ANG, angular gyrus; Aph, aphasia; Apr, apraxia; BA, Broca's area; dmPFC, dorsomedial prefrontal cortex; Dysa, dysarthria; Dysp, dysphagia; Hem, hemiplegia; IOG, inferior occipital gyrus; IPL, inferior parietal lobule; M1, primary motor cortex; MFG, middle frontal gyrus; MOG, middle occipital gyrus; mPFC, medial prefrontal cortex; MTG, middle temporal gyrus; Par, paresthesia; PCUN, precuneus; PMA, premotor area; S1, primary somatosensory cortex; SFG, superior frontal gyrus; SMA, supplementary motor area; SMG, supramarginal gyrus; TOJ, temporo‐occipital junction.

**TABLE 3:** Results of Pipeline 3.

Pipeline3		Cluster size	MNI coordinates			Brain regions	10–20 system locations	CPC coordinates	Depth
			*x*	*y*	*z*			(Pnz, Pal)	
Aph‐pos	aphn1	262	−15	54	25	SFG.L	Inferior and anterior to F3	0.22, 0.44	20.54
	aphn2	174	−38	10	49	MFG.L	Superior and anterior to C3	0.41, 0.34	19.57
	aphn3	103	−51	22	7	BA.L	Superior and posterior to F7	0.28, 0.23	21.89
	aphn4	278	−55	−55	35	ANG.L	Inferior and anterior to P3	0.71, 0.28	17.7
	aphn5	721	−4	23	60	SMA.L	Posterior to Fz	0.38, 0.48	21.65
Ata‐neg	atan1	207	8	−58	65	PCUN.R	Anterior to Pz	0.68, 0.52	22.94
	atan3	83	43	−67	27	ANG.R	Inferior to P4	0.78, 0.69	22.09
	atan4	67	22	31	50	SFG.R	Superior and posterior to F4	0.34, 0.59	20.31
Ata‐pos	atan2	56	39	−77	−18	VAC.R	Inferior and anterior to O2	0.95, 0.71	21.35
Dysa‐pos	dysan1	714	−29	50	19	MFG.L	Inferior to F3	0.22, 0.37	19.49
	dysan2	225	−53	15	0	IFGtri. L	Superior and posterior to F7	0.29, 0.20	22.89
	dysan3	114	5	16	62	SMA.R	Posterior to Fz	0.40, 0.52	23.01
	dysan4	210	52	21	0	IFGtri. R	Posterior to F8	0.25, 0.78	22.65
	dysan5	52	−6	58	3	mPFC.L	Superior and anterior to Fp1	0.14, 0.47	24.37
Hem‐neg	hemn5	78	3	65	−14	mPFC.R	Inferior and anterior to Fp2	0.08, 0.51	18.86
Hem‐pos	hemn1	62	−30	−4	60	M1.L	Superior and anterior to C3	0.47, 0.38	21.31
	hemn2	91	−56	−49	40	IPL.L	Anterior to P3	0.67, 0.27	17.51
	hemn3	127	12	21	59	SMA.R	Posterior to Fz	0.39, 0.55	22.16
	hemn4	806	27	54	15	SFG.R	Superior to Fp2	0.19, 0.62	19.8
Par‐pos	parn1	150	42	6	48	MFG.R	Anterior to C4	0.42, 0.67	20.03

*Note:* Pos, positive: brain surface regions positively correlated with the PSDs ROIs; neg, negative: brain surface regions negatively correlated with the PSD ROIs, CPC continuous proportional coordinate, L left, R right.

Abbreviations: ANG, angular gyrus; Aph, aphasia; Ata, ataxia; BA, Broca's area; Dysa, dysarthria; Hem, hemiplegia; IFGtri, inferior frontal gyrus pars triangularis; IPL, inferior parietal lobule; M1, primary motor cortex; MFG, middle frontal gyrus; Par, paresthesia; PCUN, precuneus; SFG, superior frontal gyrus; SMA, supplementary motor area; VAC, visual association cortex.

**FIGURE 2:**
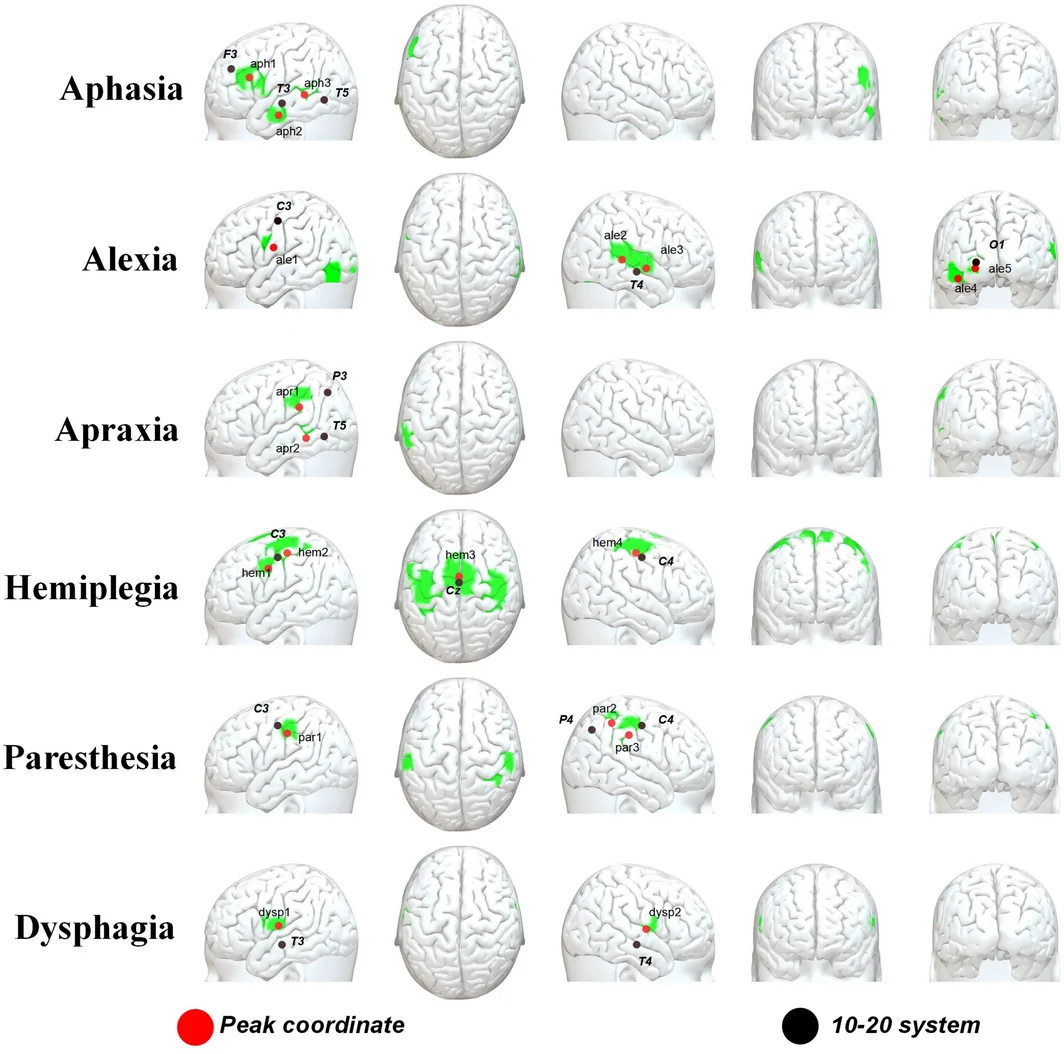
Results from Pipeline 1. Brain clusters and peak coordinates selected directly from the Neurosynth‐derived uniformity test map were shown, referenced by 10–20 system. CPC, continuous proportional coordinates; HC, healthy controls; LSM, lesion‐symptom mapping; PSD, poststroke deficits; RS‐fMRI, resting‐state functional MRI.

**FIGURE 3:**
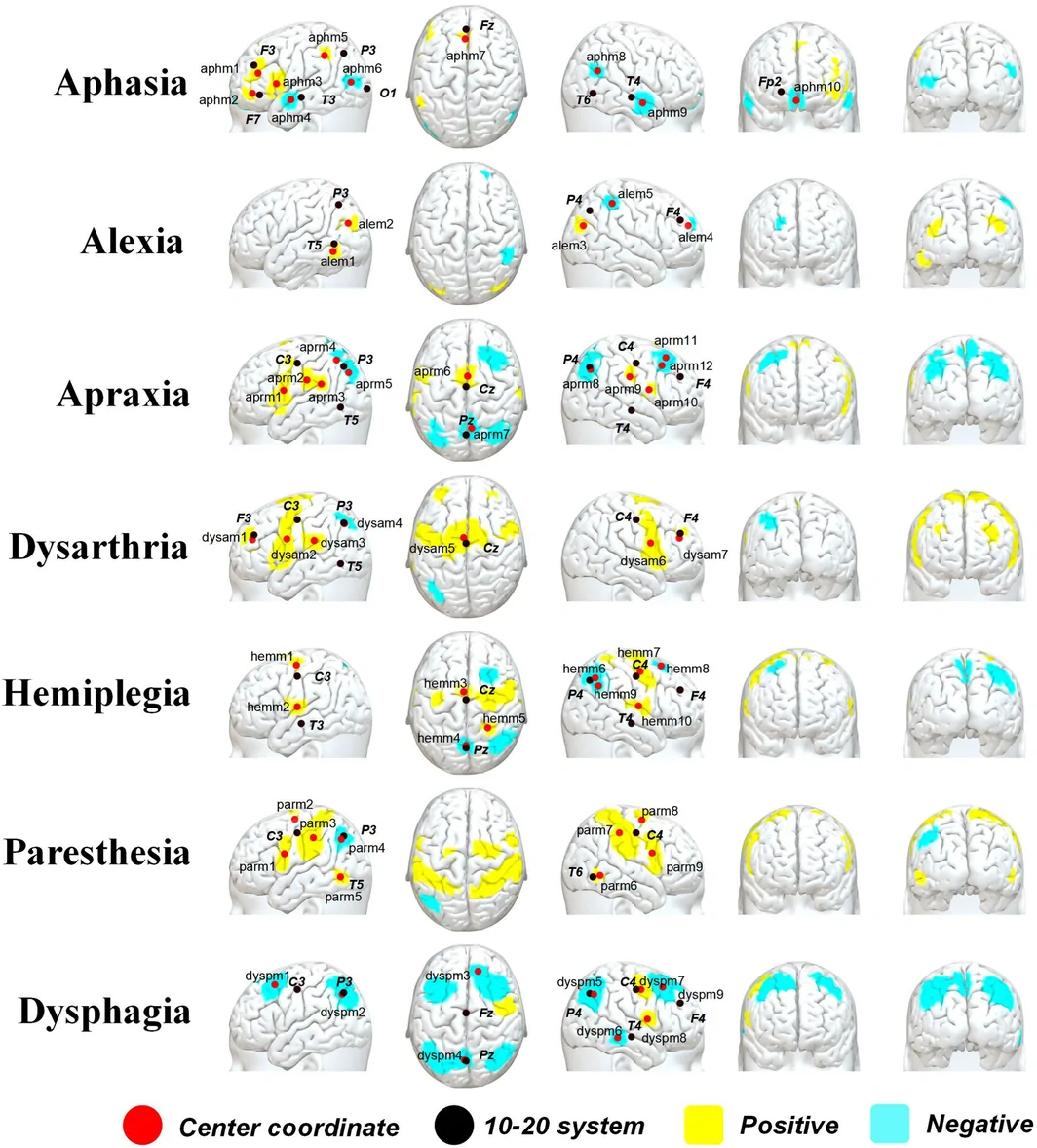
Results from Pipeline 2. Brain clusters and center coordinates explored from FC analysis based on ROIs obtained from meta‐analysis. “Positive (shown in yellow)” represents brain regions showing positive correlation; “Negative (shown in cyan)” represents brain regions showing negative correlation. FC, functional connectivity; ROI, region of interest.

**FIGURE 4:**
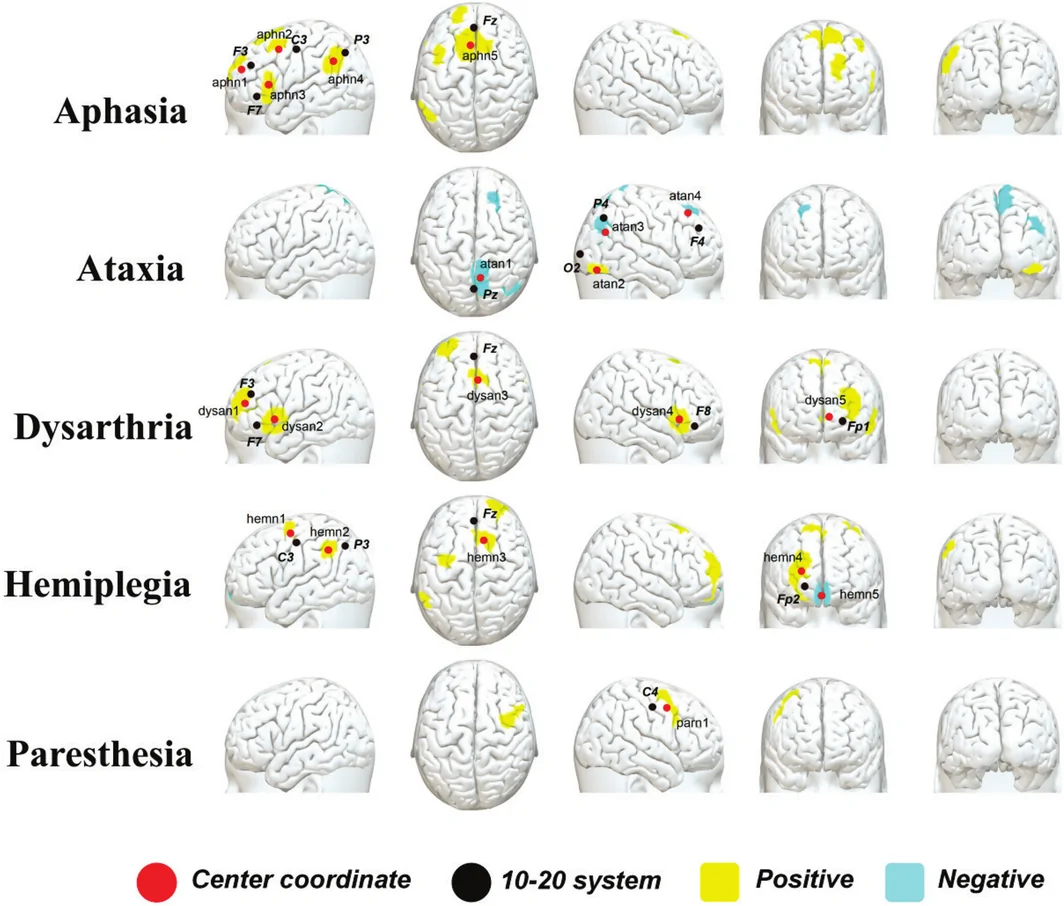
Results from Pipeline 3. Brain clusters and center coordinates explored from FC analysis based on cortical and subcortical gray matter ROIs obtained from LSM analysis (white matter regions were excluded to ensure physiological validity). “Positive (shown in yellow)” represents brain regions showing positive correlation; “Negative (shown in cyan)” represents brain regions showing negative correlation. FC, functional connectivity; LSM: lesion‐symptom mapping; ROI, region of interest.

### 3.1 Aphasia

The search terms included “stroke aphasia”, “stroke dysphasia,” and “stroke language.” Of 115 identified studies, 83 met criteria, with 128 analyses and 2808 brain coordinates.

In Pipeline 1, the Broca's area (BA), Wernicke's area (WA), and left middle temporal gyrus (MTG) were identified.

In Pipeline 2, the left MFG, BA, left inferior parietal lobule (IPL), and dorsomedial prefrontal cortex (dmPFC) were identified as regions positively correlated with aphasia ROIs, while the bilateral MTG, medial prefrontal cortex (mPFC), left temporo‐occipital junction (TOJ), and right ANG showed negative correlations.

In Pipeline 3, the BA, left superior frontal gyrus (SFG), left ANG, left MFG, and left SMA were positively correlated with aphasia lesion ROIs. No regions exhibiting significant negative correlations were identified.

### 3.2 Alexia

Of 13 studies initially retrieved for “stroke dyslexia,” “stroke alexia,” and “stroke reading,” 4 met criteria (5 analyses, 71 coordinates).

In Pipeline 1, the right posterior superior temporal gyrus (pSTG), left Visual Association Cortex (VAC), right middle superior temporal gyrus (mSTG), left primary visual cortex (PVC), and left primary motor cortex (M1)/primary somatosensory cortex (S1) were identified.

In Pipeline 2, the bilateral middle occipital gyrus (MOG), left angular gyrus (ANG), and left inferior occipital gyrus (IOG) were identified as regions positively correlated with alexia ROIs, while the right IPL and right middle frontal gyrus (MFG) showed negative correlations.

The LSM study did not identify lesion coordinates associated with alexia. Thus, Pipeline 3 yielded no results for this disorder.

### 3.3 Apraxia

Of the 16 studies initially retrieved for the terms “stroke apraxia” and “stroke hemiapraxia,” 12 met the inclusion criteria, providing 15 analyses and 269 brain coordinates.

In Pipeline 1, the left supramarginal gyrus (SMG) and left posterior middle temporal gyrus (pMTG) were identified.

In Pipeline 2, the BA/left M1, right M1, left SMG, bilateral S1, and SMA were positively correlated with apraxia ROIs, while the right ANG, left IPL, right MFG, right precuneus (PCUN), and right SFG showed negative correlations.

The LSM study did not identify lesion coordinates associated with apraxia. Thus, Pipeline 3 yielded no results for this disorder.

### 3.4 Ataxia

The search terms included “stroke ataxia” and “stroke coordination.” Of the 14 identified studies, none remained after exclusions. Therefore, no region was found from Pipeline 1 or Pipeline 2.

In Pipeline 3, the right VAC was positively correlated with ataxia lesion ROIs, while the right ANG, right SFG, and right PCUN showed negative correlations.

### 3.5 Dysarthria

After screening 8 studies using search terms “stroke dysarthria” and “stroke articulation,” only one eligible study was identified, which provided 1 analysis and 3 coordinates.

In Pipeline 1, no brain surface cluster was found on the neurosynth‐derived uniformity test map.

In Pipeline 2, the bilateral M1, bilateral MFG, left SMG, and SMA were positively correlated with dysarthria ROIs, while the left ANG showed negative correlations.

In Pipeline 3, the bilateral IFGtri, left MFG, left mPFC, and right SMA were positively correlated with dysarthria lesion ROIs. No regions exhibiting significant negative correlations were identified.

### 3.6 Hemiplegia

The search terms included “stroke hemiplegia,” “stroke paralysis,” and “stroke motor.” Of 244 identified studies, 135 met criteria, with 222 analyses and 3955 brain coordinates.

In Pipeline 1, the bilateral M1 and SMA were identified.

In Pipeline 2, the bilateral M1, bilateral S1, and SMA were positively correlated with hemiplegia ROIs, while the bilateral ANG, right PCUN, and right SFG showed negative correlations.

In Pipeline 3, the left M1, left IPL, right SMA, and right SFG were positively correlated with hemiplegia lesion ROIs, while the right mPFC showed negative correlations.

Furthermore, the supplementary validation using stroke‐patient fMRI data yielded network patterns that were consistent with those derived from the healthy cohort. For hemiplegia, the primary positive functional hubs, notably the bilateral M1 and SMA, were successfully replicated in the Pipeline 2 and 3 (Figure S1). Negative functional connectivity clusters did not reach statistical significance in this patient cohort.

### 3.7 Paresthesia

Of the 82 studies initially retrieved for the terms “stroke hypoesthesia,” “stroke paresthesia,” “stroke somatosensory”, and “stroke sensory,” 14 met the inclusion criteria, providing 25 analyses and 366 brain coordinates.

In Pipeline 1, the bilateral SMG/S1 and right superior parietal lobule (SPL) were identified.

In Pipeline 2, the bilateral TOJ, bilateral M1, bilateral S1, and bilateral premotor area (PMA) were positively correlated with paresthesia ROIs, while the left ANG showed negative correlations.

In Pipeline 3, the right MFG was positively correlated with paresthesia lesion ROIs. No regions exhibiting significant negative correlations were identified.

### 3.8 Dysphagia

Using the search terms “stroke dysphagia,” “stroke aphagia,” and “stroke swallow,” 7 studies were identified, from which 4 were included, yielding 6 analyses and 73 coordinates.

In Pipeline 1, the bilateral M1/S1 were identified.

In Pipeline 2, the right M1 was positively correlated with dysphagia ROIs, while the right MTG, bilateral IPL, bilateral PMA, and left PCUN showed negative correlations.

The LSM study did not identify lesion coordinates associated with dysphagia. Thus, Pipeline 3 yielded no results for this disorder.

## 4 Discussion

Using Neurosynth Compose, this study identified potential targets for NIBS in treating 8 common PSDs based on neuroimaging meta‐analysis, LSM, and resting‐state FC analysis. Comparing three pipelines revealed several important brain regions as potential NIBS targets. Additionally, based on the 10–20 system and the CPC coordinate system, we provided the corresponding scalp localization information for these brain targets.

### 4.1 Aphasia

We identified the BA, a core language production component, as a potential NIBS target in all pipelines. Previous studies have demonstrated that tDCS targeting BA can augment language production in poststroke aphasia [18]. The left MFG, which collaborates with the BA, is responsible for language fluency and semantic integration. Some studies have shown that rTMS applied to the left MTG can accelerate reaction times in word recognition [19]. Beyond established hubs (BA, MTG, MFG), we revealed a broader network including underexplored regions like ANG, TOJ, IPL, SMA, SFG, MFG, and mPFC. For instance, targeting the ANG or mPFC could address semantic integration deficits or cognitive control deficits [20, 21], respectively. Thus, our study moves beyond validating established targets and provides a new roadmap for exploring circuit‐based neuromodulation for aphasia.

### 4.2 Alexia

Reading relies on the integrated function of multiple brain networks, and visual attention deficits are closely associated with alexia [22]. The STG, VAC, M1, MOG, ANG, IOG, IPL, and MFG were identified as potential targets for alexia. The ANG, critically implicated in semantic processing during reading, is a core region affected in alexia [20]. The STG supports speech perception and language comprehension [23], and the MOG is involved in text decoding and visual information processing [24]. Notably, the clusters we identified predominantly localize to regions surrounding the temporo‐parieto‐occipital junction, suggesting the critical involvement of this area in alexia recovery. NIBS stimulation of the temporoparietal region can lead to faster word recognition and increased reading speed, while stimulation of the temporo‐occipital region can lead to faster word comprehension [25]. The therapeutic efficacy of these targets requires validation in future trials.

### 4.3 Apraxia

For poststroke apraxia, NIBS shows promise in enhancing motor recovery. We identified the left IPL and bilateral SMG as potential targets. Anodal tDCS targeting the left IPL improved limb praxis [26], aligning with our identification of this area. The SMG is located in the anterior region of the IPL and is involved in the neural control of goal‐directed movements. Apraxia is the inability to perform purposeful movements despite absent motor or sensory deficits. M1 and the posterior parietal cortex are key to the frontoparietal motor learning network. Anodal tDCS applied to M1 can increase cortical excitability and improve early motor skill learning [27]. These regions can be stimulated unilaterally or bilaterally on both hemispheres, depending on the lesion location, providing a comprehensive approach to apraxia treatment. Further research is needed to confirm the effectiveness of these interventions in promoting apraxia recovery.

### 4.4 Ataxia

The right ANG, right SFG, right PCUN, and right VAC were identified as potential targets for ataxia. While the cerebellum is a primary NIBS target for ataxia [28], it was not included in the findings of this study due to the use of a 25‐mm distance mask from the scalp, which excluded cerebellar regions. This does not negate the viability of the cerebellum as an intervention target but rather provides additional target options beyond the cerebellum for ataxia. Literature suggests that NIBS targeting the PCUN affects the ability to integrate visual feedback with other sensory signals to maintain balance [29]. The ANG and SFG are crucial for motor planning and may be promising targets for improving motor coordination [20, 30, 31]. The VAC is crucial for processing higher order visual information, offering potential for enhancing visuospatial integration and visual feedback ability in patients with ataxia [32]. Further research is still needed to confirm the clinical effectiveness of these interventions in promoting motor balance ability.

### 4.5 Dysarthria

Dysarthria is a common symptom after stroke. Recent studies have shown that anodal tDCS over M1 improves speech intelligibility, including rapid syllable repetition and sentence production [33]. Our results identified M1 as a core potential target, which is corroborated by recent evidence. Additionally, we found extra brain targets in the MFG, IFG, mPFC, and SMA. The SMA is responsible for motor planning and the coordination of complex movement patterns [34], while the MFG and IFG are responsible for language processing and fluency [35]. In summary, these regions provide a basis for targeted NIBS interventions to manage both motor and non‐motor symptoms of dysarthria. However, further research is needed to determine their effectiveness.

### 4.6 Hemiplegia

We identified M1, S1, SMA, ANG, SFG, mPFC, IPL, and PCUN as potential targets. The 2020 European guidelines indicate a Grade A recommendation for low‐frequency rTMS of the non‐lesioned M1 for treating upper limb paralysis in the subacute phase. Additionally, multiple studies have shown that tDCS can stimulate M1 and SMA to enhance motor function [34]. Optimal limb coordination requires the effective transmission of somatosensory information to the sensorimotor cortex. S1 damage causes somatosensory impairments, and neuromodulation targeting S1 may restore sensory feedback and improve sensorimotor integration. NIBS applied to S1 can enhance motor learning across different sensorimotor abilities [36]. Furthermore, the PCUN is primarily associated with body awareness and may improve motor execution function [37], while the ANG can improve motor ability. Similarly, the SFG plays a crucial role in higher order motor planning and complex movement coordination; targeting the SFG may facilitate cortical network reorganization and compensate for damaged motor pathways [38]. While isolated studies have explored some of these regions, our methodology concurrently implicates them as a cohesive network. This supports combining M1 stimulation with higher order regions to address complex deficits in motor planning, sensory integration, and body schema. However, further research is needed to determine its effectiveness.

Furthermore, given that our primary pipelines utilized resting‐state fMRI data from healthy subjects, one might reasonably question whether healthy connectomes can accurately reflect the reorganized networks of a poststroke brain. To directly address this, we conducted a supplementary validation specifically using resting‐state fMRI data from an independent cohort of poststroke hemiplegia patients. The results indicated that the primary positive functional hubs for hemiplegia, notably the bilateral M1 and SMA, remain spatially conserved following stroke. Significant negative functional connectivity clusters were not identified in the patient validation analysis. This absence is likely due to the small sample size of the stroke cohort, which limits the statistical power required to detect negative correlations. Utilizing a healthy connectome provides a comprehensive reference of the network topology for neuromodulation targeting. Moreover, this approach mitigates the confounding variables associated with lesion heterogeneity in stroke cohorts during group‐level functional connectivity analyses. The spatial consistency between the healthy derived positive targets and the patient derived networks supports the physiological validity of this targeting strategy for poststroke hemiplegia.

### 4.7 Paresthesia

NIBS is a promising tool for treating poststroke paresthesia (e.g., numbness and pain). In NIBS treatment of paresthesia, the M1 and S1 are the most commonly targeted regions [39]. Our findings pinpoint M1 and S1 as potential targets, which aligns with their established role as the most commonly stimulated regions for treating paresthesia. Our study offers a more refined localization within M1 and S1, which could potentially improve therapeutic outcomes. In addition, we also identified PMA, TOJ, MFG, and SMG as potential targets. The PMA integrates somatosensory feedback for motor planning. Modulating the PMA may facilitate sensorimotor integration and utilize motor networks to compensate for somatosensory impairment [40]. The TOJ processes multimodal sensory information. Targeting this region can promote multisensory integration, enabling patients to use visual spatial feedback to alleviate somatosensory deficits [41]. The SMG is involved in higher order somatic sensation processing and maintaining the body schema. Modulating the supramarginal gyrus may assist in correcting somatic misperceptions [42]. These regions provide a theoretical basis for targeted neuromodulation. Further research is required to evaluate their clinical effectiveness.

### 4.8 Dysphagia

The S1, M1, PMA, IPL, PCUN, and MTG were identified as potential targets for dysphagia. Currently, the location of the swallowing center in the cerebral cortex is not entirely clear. Some studies suggest swallowing is bilaterally innervated, with hemispheres interacting via the corpus callosum to maintain function [43]. NIBS can improve swallowing function by choosing to stimulate the unilateral or bilateral hemispheric motor cortex as needed, but there is no unified standard for the stimulation site [44, 45, 46]. Among the potential targets we identified for dysphagia are M1 and PMA, key regions in the motor cortex for planning swallowing movements [47]. Our results provide more precise localization within the motor cortex than previous broad targeting, potentially optimizing interventions. Other studies have shown that S1 is activated during swallowing, and the integrity of somatosensory function is an important guarantee for the recovery of swallowing function [48, 49]. Activation of the bilateral IPL is associated with oromotor and sensory functions and may be a characteristic of bilateral movements such as swallowing [50]. This comprehensive network view provides a radical departure from the dominant motor‐centric approach and opens up multiple new targets for this complex condition. However, further research is needed to explore the therapeutic potential of these targets and to optimize intervention strategies for this complex condition.

## 5 Recommendations Across Pipelines and PSDs

While the optimal analytical pipeline remains unknown, targets identified consistently across multiple pipelines, such as BA for aphasia or the M1 for hemiplegia, demonstrate high convergence across meta‐analytic, structural, and functional data analyses. We propose these overlapping regions as primary targets for clinical application. Conversely, targets identified in a single pipeline may serve as secondary or alternative options for patients who do not respond to primary stimulation protocols or when primary targets are contraindicated due to specific lesion topographies.

While this study evaluated potential targets for each PSD in isolation, clinical reality often involves comorbid presentations. Our findings highlight overlapping brain regions, such as the ANG and SMA, which emerged as targets across multiple PSDs. Clinicians treating patients with concurrent PSDs might prioritize these overlapping regions to simultaneously modulate multiple impaired networks.

## 6 Limitations

This study has several limitations. First, relying on Neurosynth Compose means identified cortical targets originate from literature meta‐analysis up to our initial search date (July 26, 2024). Given the rapid pace of neuromodulation research, these networks may evolve. To address this, we conducted a supplementary scoping search using the original keywords for literature published between August 2024 and April 2026. This search revealed minimal new additions for most deficits, yielding 0 new eligible studies for apraxia, 2 for alexia and dysarthria, 5 for dysphagia and ataxia, and 6 for paresthesia. While aphasia and hemiplegia yielded 16 and 27 new studies respectively, a review of these recent publications confirmed they remain consistent with our primary identified targets. Therefore, we found no evidence that recent literature contradicts or substantially alters the validity of the current meta‐analytic targets. Nevertheless, future data updates and refined personalized protocols are needed to continuously improve target selection. Second, while some identified targets are consistent with previous brain stimulation research, others remain theoretical and require clinical validation. Third, due to limited literature or constraints of the NIHSS assessment, some PSDs yielded no results in certain pipelines. Future studies may address this with updated literature and domain‐specific functional assessments. Fourth, the optimal pipeline remains unknown, though a comprehensive understanding of these regions map aid clinical target selection. Fifth, although we restricted the meta‐analytic ROIs to voxels overlapping with the original Neurosynth uniformity test maps to improve functional specificity, the use of 6‐mm‐radius spherical ROIs remains a limitation, as such uniform spheres do not respect anatomical boundaries and may introduce signal contamination from adjacent gyri. Sixth, although a healthy connectome was utilized for target identification and validated using hemiplegia patient data, other PSDs that have not been validated still require further investigation in stroke patients. Finally, specific treatment parameters (e.g., intensity, frequency, and duration) are beyond this study's scope, warranting further research. Despite these limitations, this study establishes a systematic, neuroimaging‐guided framework for NIBS target identification, offering valuable insights for optimizing evidence‐based interventions for neurological disorders.

## 7 Conclusion

Conclusively, by combining neurosynth‐based meta‐analysis, LSM, and resting‐state FC analysis, we have identified potential NIBS targets for different PSDs. Although these targets require further validation, these findings offer new insights for the future application of NIBS in the treatment of patients with PSDs.

## Author Contributions

**Sitong Yuan:** conceptualization, methodology, writing – original draft; **Yue Yang:** data curation, formal analysis; **Yi Han:** data curation, investigation; **Ying Gao:** conceptualization, writing – review and editing; **Jingling Chang:** conceptualization, writing – review and editing; **Huize Lin:** methodology; **Jinna Yu:** methodology, supervision; **Binlong Zhang:** conceptualization, supervision, writing – review and editing. All authors reviewed and edited manuscript revisions and approved the final version of the paper.

## Funding

This study was supported by the Beijing Municipal Health Commission (Beijing High Level Innovation and Entrepreneurship Talent Support Program, project No. G202534224), China Association for Science and Technology (Young Elite Scientists Sponsorship Program by CAST, project No. 2022QNRC001), National Natural Science Foundation of China (National Natural Science Foundation of China, project No. 82205299), China Academy of Chinese Medical Sciences (Fundamental Research Funds for the Central public welfare research institutes, project No. ZZ14‐YQ‐017),China Academy of Chinese Medical Sciences (High Level Chinese Medical Hospital Promotion Project, project No. HLCMHPP2023003), China Academy of Chinese Medical Sciences (The Escort Project of Guang'anmen Hospital, China Academy of Chinese Medical Science‐Backbone Talent Cultivation Project, project No. GAMHH9324024), Beijing Municipal Administration of Traditional Chinese Medicine (Famous Doctor Inheritance Workstation of Liu Baoyan, project No. 2025‐SZ‐C‐95), and China Academy of Chinese Medical Sciences (Scientific and technological innovation project of China Academy of Chinese Medical Sciences, project No. CI2021A03313).

## Ethics Statement

The data used in this study were sourced from publicly accessible databases, and thus no additional ethical approval was required. All the original studies related to the databases had already obtained the necessary ethical approvals and participants' consents prior to the investigations.

## Conflicts of Interest

The authors declare no conflicts of interest.

## Supporting information


**Table S1:** Regions of interest obtained from lesion‐symptom mapping.
**Table S2:** Literature screening and data extraction in neurosynth‐based meta‐analysis.
**Table S3:** Regions of interest obtained from neurosynth‐based meta‐analysis.
**Figure S1:** Validation of functional connectivity networks for hemiplegia using resting‐state fMRI data from poststroke patients.

## Data Availability

The data that support the findings of this study are available from the corresponding author on reasonable request.
