# Supplementary material for: Potential Locations for Noninvasive Brain Stimulation in Treating Poststroke Deficits: Exploration From Multimodal Neuroimaging
Source: CNS Neurosci Ther. 2026 Oct 29;32(11):e71229. doi: 10.1002/cns.71229 (PMC13717485; doi:10.1002/cns.71229)
Supplement: Supplementary file 1 [file CNS-32-e71229-s001.docx]

**Supplementary material**

**Supplementary Methods**

**Supplementary Table 1.** Regions of interest obtained from lesion-symptom mapping.

**Supplementary Table 2.** Literature screening and data extraction in neurosynth-based meta-analysis.

**Supplementary Table 3.** Regions of interest obtained from neurosynth-based meta-analysis.

**Supplementary Figure 1.** Validation of functional connectivity networks for hemiplegia using resting-state fMRI data from post-stroke patients.

**Supplementary Methods**

**Participants and MRI Acquisition**

During MRI collection, subjects were instructed to lie down, close their eyes, and rest without thinking about anything specific without falling asleep. 3D-T1 was acquire by a magnetization-prepared rapid gradient echo (MPRAGE) sequence with the following parameters: repetition time=1,900 ms, echo time=2.52 ms, inversion time=900 ms, flip angle=90 degrees, resolution matrix=256×256, slices=176, thickness =1.0 mm, and voxel size=1×1×1 mm3. An 8-minute RS-fMRI scan with 242 images was acquired using gradient echo echo-planar-imaging (GRE-EPI) sequences with the following parameters: slices=32, repetition time/echo time=2000/30 ms, flip angle=90, field of view=220×220 mm, thickness/slice gap=3/1 mm, and voxel size=3.4×3.4×4 mm3.

**Supplementary Table 1.** Regions of interest obtained from lesion-symptom mapping

| **NIHSS score** | **Region** | **Coordinate (x,y,z)** |
| --- | --- | --- |
| **NIHSS 5a. Left arm** | Frontal Pole,R | (35,46,4) |
|  | Frontal Orbital Cortex,R | (29,23,-15) |
|  | Temporal Pole,R | (41,13,-28) |
|  | Inferior fronto-occipital fasciculus R | (35,32,-1) |
|  | Superior longitudinal fasciculus R | (49,-2,25) |
|  | Uncinate fasciculus R | (33,18,-19) |
| **NIHSS 5b. Right arm** | Frontal Pole,L | (-27,50,9) |
|  | Postcentral Gyrus,L | (-40,-29,56) |
|  | Precentral Gyrus,L | (-25,-18,62) |
|  | Corticospinal tract L | (-22,-28,57) |
|  | Forceps minor | (-16,46,6) |
|  | Superior longitudinal fasciculus L | (-38,-29,45) |
| **NIHSS 6a. Left leg** | Frontal Pole,R | (27,46,14) |
|  | Temporal Pole,R | (35,14,-32) |
|  | Superior Frontal Gyrus,R | (14,24,56) |
|  | Forceps minor | (14,48,13) |
|  | Inferior fronto-occipital fasciculus R | (37,31,2) |
|  | Uncinate fasciculus R | (32,20,-23) |
| **NIHSS 6b. Right leg** | Postcentral Gyrus,L | (-36,-29,54) |
|  | Precentral Gyrus,L | (-22,-20,60) |
|  | Frontal Pole,L | (-26,50,14) |
|  | Corticospinal tract L | (-20,-28,54) |
|  | Superior longitudinal fasciculus L | (-40,-19,35) |
|  | Forceps minor | (-15,44,6) |
| **NIHSS 7. Limb ataxia** | Lateral Occipital Cortex,inferior division,R | (39,-81,-27) |
|  | Inferior Temporal Gyrus,temporooccipital part,L | (-50,-59,-27) |
|  | Temporal Occipital Fusiform Cortex,L | (-48,-61,-27) |
|  | Corticospinal tract R | (19,-41,-37) |
|  | Corticospinal tract L | (-37,-61,-44) |
|  | Anterior thalamic radiation L | (-34,-64,-47) |
|  | Cerebellar Left VIIIa | (-29,-51,-52) |
|  | Cerebellar Right Crus II | (34,-76,-46) |
|  | Cerebellar Left Crus II | (-35,-66,-48) |
| **NIHSS 8. Sensation** | Superior Frontal Gyrus,R | (21,10,62) |
|  | Middle Frontal Gyrus,R | (37,13,55) |
|  | Precentral Gyrus,R | (51,-2,44) |
|  | Corticospinal tract R | (16,-24,5) |
|  | Anterior thalamic radiation R | (13,-21,13) |
|  | Superior longitudinal fasciculus R | (58,-3,35) |
| **NIHSS 9. Language** | Frontal Pole,L | (-29,53,11) |
|  | Temporal Pole,L | (-46,11,-25) |
|  | Middle Temporal Gyrus,posterior division,L | (-61,-23,-12) |
|  | Inferior longitudinal fasciculus L | (-47,4,-22) |
|  | Superior longitudinal fasciculus L | (-57,-25,15) |
|  | Uncinate fasciculus L | (-38,18,-19) |
| **NIHSS 10. Dysarthria** | Parahippocampal Gyrus,anterior division,L | (-18,-17,-25) |
|  | Frontal Orbital Cortex,L | (-31,31,-4) |
|  | Insular Cortex,L | (-33,12,-8) |
|  | Inferior fronto-occipital fasciculus L | (-33,32,0) |
|  | Uncinate fasciculus L | (-33,19,-9) |
|  | Corticospinal tract L | (-14,-18,-17) |

NIHSS=National Institutesof Health Stroke Scale; R=right; L=left; OR=odds ratio. (data were extracted from Ding et al.^1^).

**Supplementary Table 2.** Literature screening and data extraction in neurosynth-based meta-analysis.

| **PSD** | **Retrieved studies** | **Excluded studies** | **Included studies** | **Number of analyses** | **Coordinates** |
| --- | --- | --- | --- | --- | --- |
| **Aphasia** | 115 | 32 | 83 | 128 | 2808 |
| **Alexia** | 13 | 9 | 4 | 5 | 71 |
| **Apraxia** | 16 | 4 | 12 | 15 | 269 |
| **Ataxia** | 14 | 14 | 0 | 0 | 0 |
| **Dysarthria** | 8 | 7 | 1 | 1 | 3 |
| **Hemiplegia** | 244 | 109 | 135 | 222 | 3955 |
| **Paresthesia** | 82 | 68 | 14 | 25 | 366 |
| **Dysphagia** | 7 | 3 | 4 | 6 | 73 |

An analysis represents a single statistical contrast between any number of groups/conditions. PSD=post-stroke deficit.

**Supplementary Table 3.** Regions of interest obtained from neurosynth-based meta-analysis.

| **PSD** | **MNI coordinates** | | |
| --- | --- | --- | --- |
|  | **x** | **y** | **z** |
| **Aphaisa** | -46 | 22 | 20 |
|  | -46 | -24 | 4 |
|  | -42 | 8 | 24 |
|  | -54 | -10 | -24 |
|  | -6 | 28 | 42 |
|  | 2 | 28 | 40 |
|  | -10 | 28 | 36 |
|  | -2 | 26 | 40 |
|  | 32 | 20 | -2 |
|  | 38 | 24 | 4 |
|  |  |  |  |
| **Alexia** | -48 | -52 | -18 |
|  | -38 | -40 | -18 |
|  | -38 | -46 | -24 |
|  |  |  |  |
| **Apraxia** | -50 | -54 | 16 |
|  | -58 | -8 | 16 |
|  | -32 | -30 | 36 |
|  | -28 | 2 | -8 |
|  | 0 | 18 | 40 |
|  | 22 | 30 | 36 |
|  |  |  |  |
| **Dysarthria** | -26 | -2 | 10 |
|  | -2 | -26 | -4 |
|  |  |  |  |
| **Paresthesia** | 48 | -22 | 38 |
|  | 50 | -18 | 26 |
|  | 42 | -34 | 40 |
|  | 50 | -26 | 36 |
|  | -58 | -24 | 42 |
|  | 50 | 6 | 18 |
|  | 62 | -32 | -10 |
|  |  |  |  |
| **Hemiplegia** | 2 | -10 | 54 |
|  | 36 | -22 | 56 |
|  | -4 | -4 | 56 |
|  | 38 | -18 | 54 |
|  | -28 | -62 | -22 |
|  | 16 | -54 | -20 |
|  | -20 | -56 | -20 |
|  | -24 | -68 | -26 |
|  | -18 | -16 | 6 |
|  | -18 | -14 | 14 |
|  |  |  |  |
| **Dysphagia** | 54 | -2 | 14 |
|  | -2 | -4 | 54 |
|  | -10 | -64 | -18 |
|  | 12 | -62 | -30 |

MNI Montreal Neurological Institute. PSD=post-stroke deficit.

**Supplementary Figure 1. Validation of functional connectivity networks for hemiplegia using resting-state fMRI data from post-stroke patients.** Brain clusters and center coordinates were identified via seed-to-voxel FC analysis using an independent cohort of post-stroke patients with hemiplegia. Pipeline 2 displays networks based on ROIs obtained from the neuroimaging meta-analysis. Pipeline 3 displays networks based on ROIs obtained from the LSM analysis. 'Positive (shown in yellow)' represents brain regions showing positive correlation; Red dots represent cluster center coordinates, and black dots represent the 10-20 system locations. FC: functional connectivity; ROI: region of interest; LSM: lesion-symptom mapping.

**References**

1. Ding L, Liu H, Jing J, et al. Lesion Network Mapping for Neurological Deficit in Acute Ischemic Stroke. Ann. Neurol. 2023;94(3):572–584.
